# Cancer Cells Acquire Mitotic Drug Resistance Properties Through Beta I-Tubulin Mutations and Alterations in the Expression of Beta-Tubulin Isotypes

**DOI:** 10.1371/journal.pone.0012564

**Published:** 2010-09-03

**Authors:** Chun Hei Antonio Cheung, Su-Ying Wu, Tian-Ren Lee, Chi-Yen Chang, Jian-Sung Wu, Hsing-Pang Hsieh, Jang-Yang Chang

**Affiliations:** 1 National Institute of Cancer Research, National Health Research Institutes (NHRI), Tainan, Taiwan, Republic of China; 2 Division of Biotechnology and Pharmaceutical Research, National Health Research Institutes (NHRI), Zhunan, Taiwan, Republic of China; 3 Division of Hematology and Oncology, Department of Internal Medicine, National Cheng Kung University Hospital, Tainan, Taiwan, Republic of China; Sun Yat-Sen University, China

## Abstract

**Background:**

Anti-mitotic compounds (microtubule de-stabilizers) such as vincristine and vinblastine have been shown clinically successful in treating various cancers. However, development of drug-resistance cells limits their efficacies in clinical situations. Therefore, experiments were performed to determine possible drug resistance mechanisms related to the application of anti-mitotic cancer therapy.

**Principal Findings:**

A KB-derived microtubule de-stabilizer-resistant KB-*L30* cancer cell line was generated for this study. KB-*L30* cells showed cross-resistance to various microtubule de-stabilizers including BPR0L075, vincristine and colchicine through multiple-drug resistant (MDR)-independent mechanisms. Surprisingly, KB-*L30* cells showed hyper-sensitivity to the microtubule-stabilizer, paclitaxel. Results of the RT-PCR analysis revealed that expression of both class II and III β-tubulin was down-regulated in KB-*L30* cells as compared to its parental KB cancer cells. In addition, DNA sequencing analysis revealed six novel mutation sites present in exon four of the βI-tubulin gene. Computational modeling indicated that a direct relationship exists between βI-tubulin mutations and alteration in the microtubule assembly and dynamic instability in KB-*L30* cells and this predicted model was supported by an increased microtubule assembly and reduced microtubule dynamic instability in KB-*L30* cells, as shown by Western blot analysis.

**Conclusions and Significance:**

Our study demonstrated that these novel mutations in exon four of the βI-tubulin induced resistance to microtubule de-stabilizers and hyper-sensitivity to microtubule stabilizer through an alteration in the microtubule assembly and dynamics in cancer cells. Importantly, the current study reveals that cancer cells may acquire drug resistance ability to anti-mitotic compounds through multiple changes in the microtubule networks. This study further provided molecular information in drug selection for patients with specific tubulin mutations.

## Introduction

Microtubules are protein filaments of the cytoskeleton composed of α-tubulin and β-tubulin molecules. In cells, microtubule filaments rapidly alternate between phases of growth and shrinkage (dynamic instability) during cell cycle [1]. Since microtubules play crucial roles in the regulation of the mitotic apparatus, disruption of microtubules can induce cell cycle arrest in M phase, the formation of abnormal mitotic spindles, and finally triggering of signals for apoptosis. The discovery that the cytotoxic activity of various compounds is through the interference with the mitotic spindle apparatus has attracted much attention within the past two decades, and microtubules have become an attractive pharmacological target for anticancer drug discovery. Anti-mitotic compounds such as vincristine, vinblastine (microtubule-destabilizing *Vinca* alkaloid) and paclitaxel (microtubule-stabilizing taxane) have been developed to target cancers clinically [2], [3]. Although the taxanes and *Vinca* alkaloids are effective for the management of different malignancies, their potential is limited by the development of multidrug resistance (MDR) [4], [5]. MDR is multi-factorial, with one pathway leading to resistance mediated by the over-expression of transmembrane efflux pumps, namely, the *M*r 170,000 P-glycoprotein (P-gp170/MDR) and the multidrug resistance protein (MRP) [5], [6]. Besides the expression of MDR, additional mechanisms of resistance to anti-mitotic drugs have also been described previously. These include changes in tubulin isotype expression and mutations in the tubulin gene [7], [8]. Cells containing mutations such as D45Y, S172A, D197N, D224N, S234N, L240I and K350N in the class I beta-tubulin have been found resistant to colchicine and *Vinca* alkaloid [7]. On the other hand, cells containing mutations such as P173A, Q292E and Y422C in the class I beta-tubulin have been found resistant to epothilone (microtubule stabilizing agent) [9]. Interestingly, over-expression of βIII-tubulin has been shown in paclitaxel-resistant cells [10], [11], [12]. However, combined changes (alternations in in tubulin isotype expression and mutations in the β-tubulin gene) in the microtubule networks are seldom demonstrated in the anti-mitotic drug resistance cancer cells.

In this study, a microtubule de-stabilizer-resistant cancer cell line was used to investigate novel changes present in cells that were able to induce resistance to anti-mitotic compounds. KB-*L30* is a KB-derived BPR0L075 (microtubule de-stabilizer)-resistant cancer cell line. Our published study revealed that KB-*L30* cells over-expressed survivin, leading to the stabilization of microtubule networks and resulting in resistance to microtubule de-stabilizing compounds [13]. However, down-regulation of survivin only partially restored the drug-sensitivity to microtubule de-stabilizers colchicine and BPR0L075, suggesting that additional drug-resistant mechanism is present in this cell line [13]. Here, we investigated additional mechanisms that may be responsible for drug-resistance to microtubule de-stabilizers in KB-*L30* cells.

## Results

### KB-*L30* cells show drug-resistance to microtubule de-stabilizers and hyper-sensitivity to microtubule stabilizer

**Figure 1:**
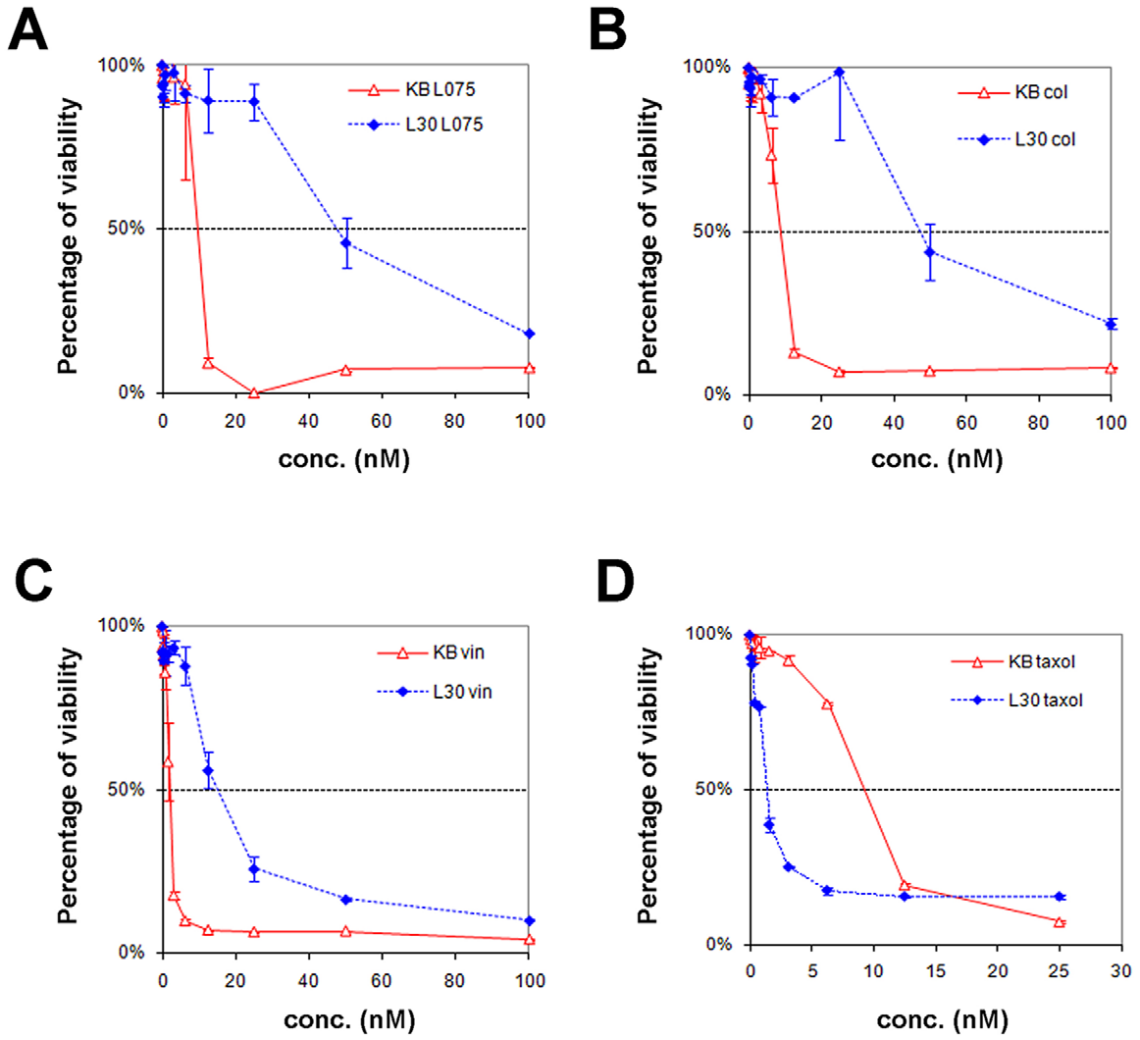
KB-*L30* cells show resistant to microtubule de-stabilizers and hyper-sensitive to microtubule stabilizer. KB and KB-*L30* cells were treated with various concentrations of BPR0L075 (A), colchicine (B), vincristine (C) and paclitaxel (D) for 3 days and cell viability was measured by MTT cell viability assay.

A KB-derived BPR0L075-resistant cancer cell line, KB-*L30*, was generated in our laboratory. In brief, KB-*L30* was a monoclonal cell line selected for resistance by continual exposure of the parental cancer cell line, KB, to increasing concentrations of the microtubule de-stabilizing compound, BPR0L075. KB-*L30* cells are cultured in the medium with 30 nM of BPR0L075 to maintain its drug resistant characteristic. This specific drug-resistant cancer cell line is 6-fold more resistant to BPR0L075 as compared to its parental cells (Table 1) (Figure 1A). In addition, KB-*L30* cells exhibit cross-resistance with another microtubule de-stabilizing agents, colchicine (7-fold resistant) and vincristine (7-fold resistant) (Table 1) (Figure 1B and C). Surprisingly, this drug-resistant cancer cell line is 5-fold more sensitive to the microtubule stabilizing agent paclitaxel as compare to KB cells (Table 1) (Figure 1D).

**Table 1:** Characterization of KB and KB-*L30* cells.

		IC50 conc.	(nM)	
	Anti-mitotic effect	KB	KB-*L30*	Differences
**BPR0L075**	*de-stabilizing*	7.2±3.3	42.4±7.4	6-fold ↑
**Vincristine**	*de-stabilizing*	2.7±1.1	19.5±6.4	7-fold ↑
**Colchicine**	*de-stabilizing*	9.8±1.5	64.0±23.8	7-fold ↑
**Paclitaxel**	*stabilizing*	10.0±1.1	1.6±0.6	**5-fold ↓**
**Doubling time**		26 hours	30 hours	

Cells were treated with various drugs for 3 days and cell viability was analyzed by MTT cell viability assay.

### Multiple-drug resistant protein (MDR) is not present in KB-*L30* cells

**Figure 2:**
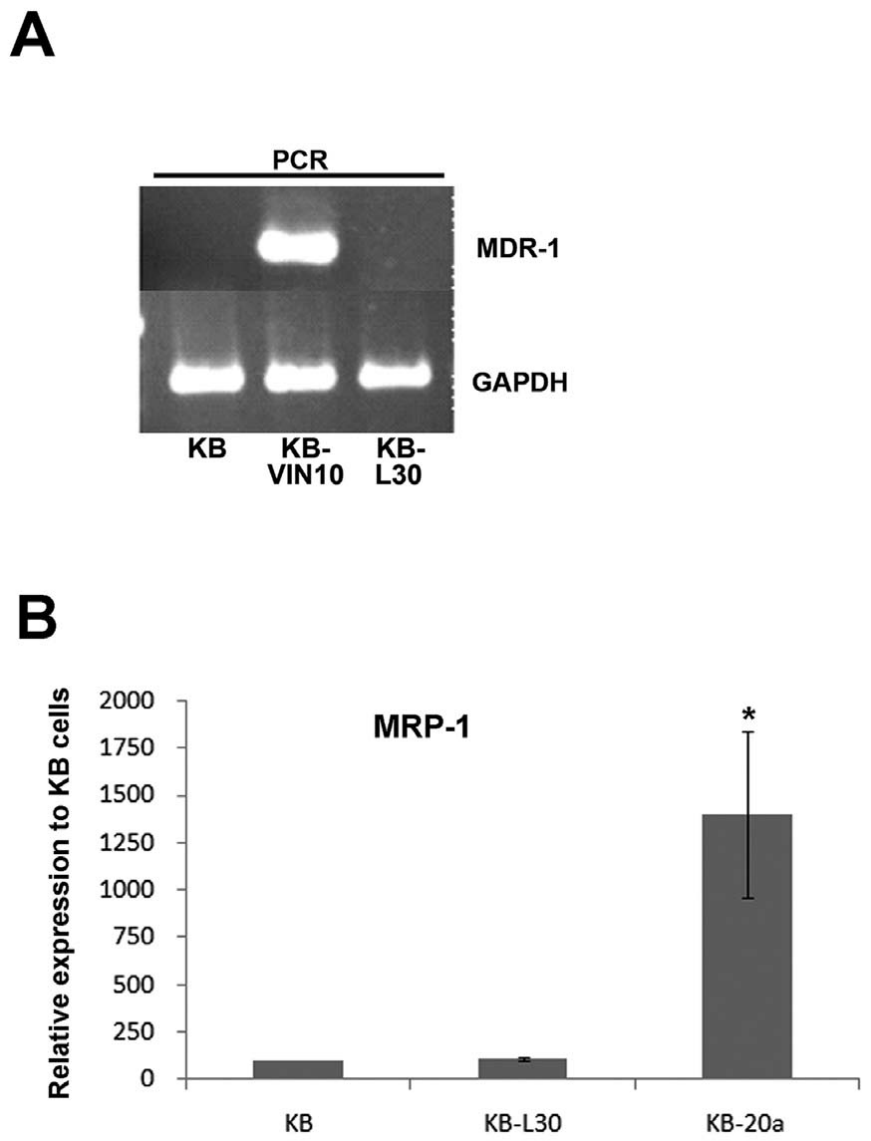
KB-*L30* cells exhibit MDR/MRP-independent drug-resistant properties. The expression of MDR-1 in KB, KB-*L30* and KB-VIN10 cells was analyzed by RT-PCR (A). The expression of MRP-1 in KB, KB-*L30* and KB-20a cells was analyzed by real-time PCR (B). GAPDH was used as an internal control in both RT-PCR and real time PCR analyzes.

Previous study demonstrated that the microtubule de-stabilizing compound BPR0L075 was equally effective in both MDR/MRP negative and positive cancer cells [14], indirectly suggesting that the BPR0L075-resistance KB-*L30* cells exhibit drug resistance properties through MDR/MRP-independent mechanism. To further support the above findings, RT-PCR was performed to determine whether KB-*L30* cells over-expressed MDR-1. RT-PCR analysis revealed that neither KB nor KB-*L30* cells express MDR-1 (Figure 2A). In contrast, MDR-1 was expressed in the vincristine-resistant cancer cell line, KB-VIN10 (positive control) (Figure 2A). Furthermore, results from the quantitative real-time PCR revealed that another important drug efflux pump, MPR-1, was not over-expressed in KB-*L30* cells as compared to its parental KB cells (Figure 2B). In contrast, MRP-1 was over-expressed in the positive control cell line, KB-20a (Figure 2B). Taken together, these results suggested that KB-*L30* cells induced resistance to microtubule de-stabilizers through MDR-independent mechanisms.

### KB-*L30* cells show alterations in beta tubulin isotype expression

**Figure 3:**
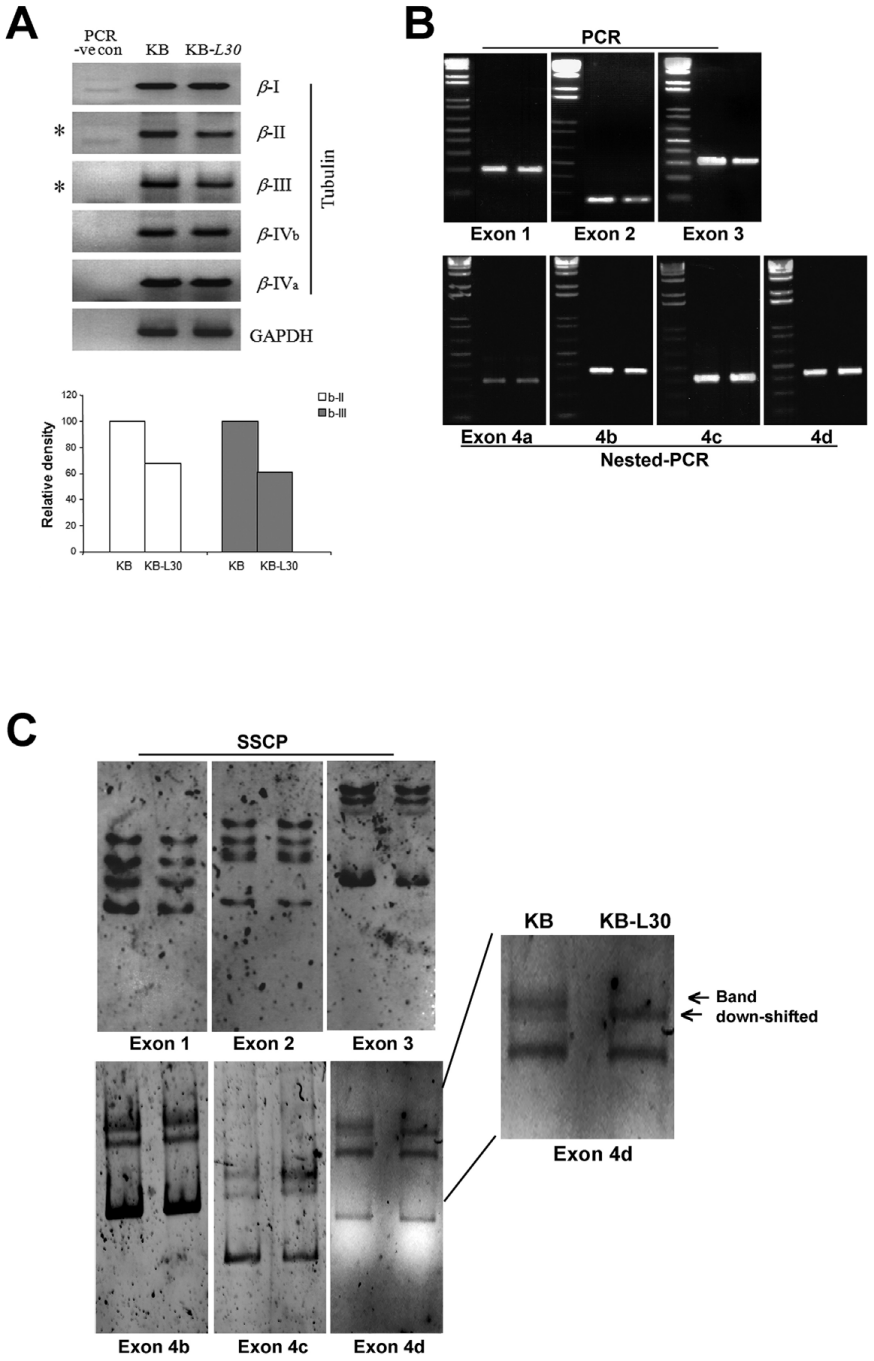
KB-*L30* cells exhibit alterations in the expression of β-tubulin isotypes and mutations in the class III β-tubulin gene. The expression of different β-tubulin isotypes in KB and KB-*L30* cells was analyzed by RT-PCR. GAPDH was used as an internal control (A). RT-PCR and nested-PCR analyzes of exons 1, 2, 3 and 4 in the class III β-tubulin gene of KB and KB-*L30* cells. First lane, KB cells and second lane, KB-*L30* cells (B). PCR-SSCP analyzes of exons 1, 2, 3 and 4 in the class III β-tubulin gene of KB and KB-*L30* cells. First lane, KB cells and second lane, KB-*L30* cells (C).

Since KB-*L30* cells induced BPR0L075 and cochicine-resistance through MDR/ MRP-independent mechanism, other drug-resistance mechanisms has been investigated. It has been widely demonstrated that alterations of the expression of β-tubulin isotypes at the mRNA level are related to the induction mitotic drug-resistance in cancer cells [15], [16]. Here, expression of β-tubulin isotypes in KB and KB-*L30* cells was analyzed by RT-PCR. At the mRNA level, the expression of class II and III β-tubulin isotypes was reduced in KB-*L30* cells as compared to the microtubule de-stabilizers sensitive KB cells (Figure 3A). The amount of class II and III β-tubulin isotypes expressed in KB-*L30* cells was reduced by 35% and 40% as compared to its parental cells. In contrast, there was no significant difference in the expression of class I, IV_a_ and IV_b_ β-tubulin isotypes between the two cell lines (Figure 3A).

### KB-*L30* cells exhibit multiple mutations in exon four of the βI-tubulin gene

The contribution of β-tubulin point mutations in inducing drug resistance to various anti-mitotic compounds was also described previously [7], [8], [17]. Since class I β-tubulin represents ∼90% of the total intracellular pool in cells, the gene sequence of βI-tubulin of KB-*L30* cells was analyzed. First, RT-PCR was performed to determine if any large scale sequence insertion or deletion were present in exon 1, 2, 3 and 4 of the βI-tubulin gene of KB-*L30* cells. Results of RT-PCR and gel electrophoresis revealed no significant differences in the molecular size (length) of any of the exons of βI-tubulin gene between KB-*L30* and KB cells (Figure 3B). This result indicated that large scale sequence insertion or deletion was not present in the βI-tubulin gene of KB-*L30* cells. We further determined the presence of single point mutation in KB-*L30* cells by performing Single-strand Conformation Polymorphism (SSCP) analysis. SSCP is the electrophoretic separation of single-stranded nucleic acids based on subtle differences in sequence (often a single base pair), resulting in a different secondary structure and a measurable difference in mobility through a gel. Here, results of SSCP analysis of the βI-tubulin genomic DNA showed no mobility band shift of exon 1, 2 and 3 of KB-*L30*, as compared to that of KB cells (Figure 3C). Surprisingly, SSCP analysis revealed a pattern shift in exon 4 of βI-tubulin in KB-*L30* cells.

**Figure 4:**
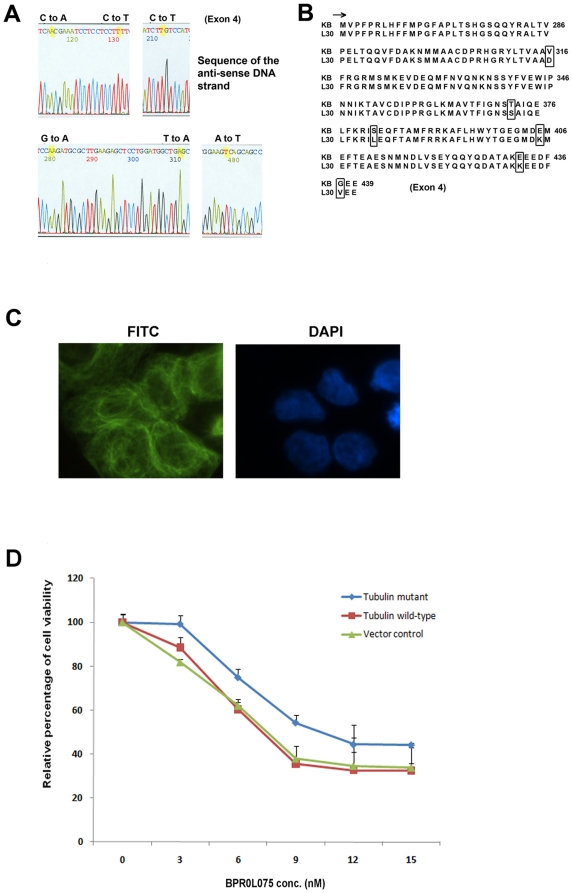
Mutations in exon 4 of βIII-tubulin interfere with the sensitivity of BPR0L075 in KB-*L30* cells. DNA sequencing of the βI-tubulin genomic DNA reveals six point mutations present in exon 4 (A). Translated amino-acid sequence of the mutated exon 4 of the βI-tubulin gene (B). The expression of the mutated βI-tubulin protein and its incorporation in the microtubule networks were revealed by immuno-fluorescent microscopy. Cells were probed with anti-βI-tubulin antibody (C). Transfection of the mutated βI-tubulin gene into KB cells reduced the drug sensitivity to BPR0L075 in vitro (D).

DNA sequencing was performed to re-confirm the above results. Consistent with the results of both RT-PCR and SSCP analysis, DNA sequencing of the βI-tubulin of KB-*L30* cells revealed no mutation present in its exon 1, 2 and 3 regions (data not shown). Interestingly, DNA sequencing revealed multiple mutations present in exon 4 of βI-tubulin of KB-*L30* cells. The sequencing data are the results of a minimum of two experiments. Six point mutations were found at nucleotide 947 from T to A (sense), 1114 from A to T, 1145 from C to T, 1213 from G to A, 1294 from G to A and 1310 from G to T (Figure 4A). These mutations correspond to amino-acid mutations V316D, T372S, S382L, E405K, E432K and G437K (Figure 4B).

Immunofluorescence staining was performed to determine whether the mutated βI-tubulin gene did translate into proteins that were subsequently incorporated into the microtubule networks in KB-*L30* cells. KB-*L30* cells were stained with anti-βI-tubulin antibody and the FITC-conjugated secondary antibody. Results of the immunofluorescent microscopy revealed that the mutated βI-tubulin protein was present as part of the microtubule networks (Figure 4C). To demonstrate that tubulin mutations were indeed responsible for causing the BPR0L075-resistant phenotype, gene transfection and over-expression of the βI-tubulin mutant that contains the above mutations was performed in KB cells. MTT cell viability assay revealed that KB cells transfected with the mutated βI-tubulin showed increased resistance to BPR0L075 (IC_50_ 11 nM) as compared to the cells transfected with the control empty plasmid (IC_50_ 7 nM) and wild-type βI-tubulin (IC_50_ 7 nM) (Figure 4D). Thus, the expression of the mutated βI-tubulin did play a role in drug sensitivity to BPR0L075 in KB cells.

### Computer modeling studies for βI-tubulin mutations in BPR0L075 resistance

**Figure 5:**
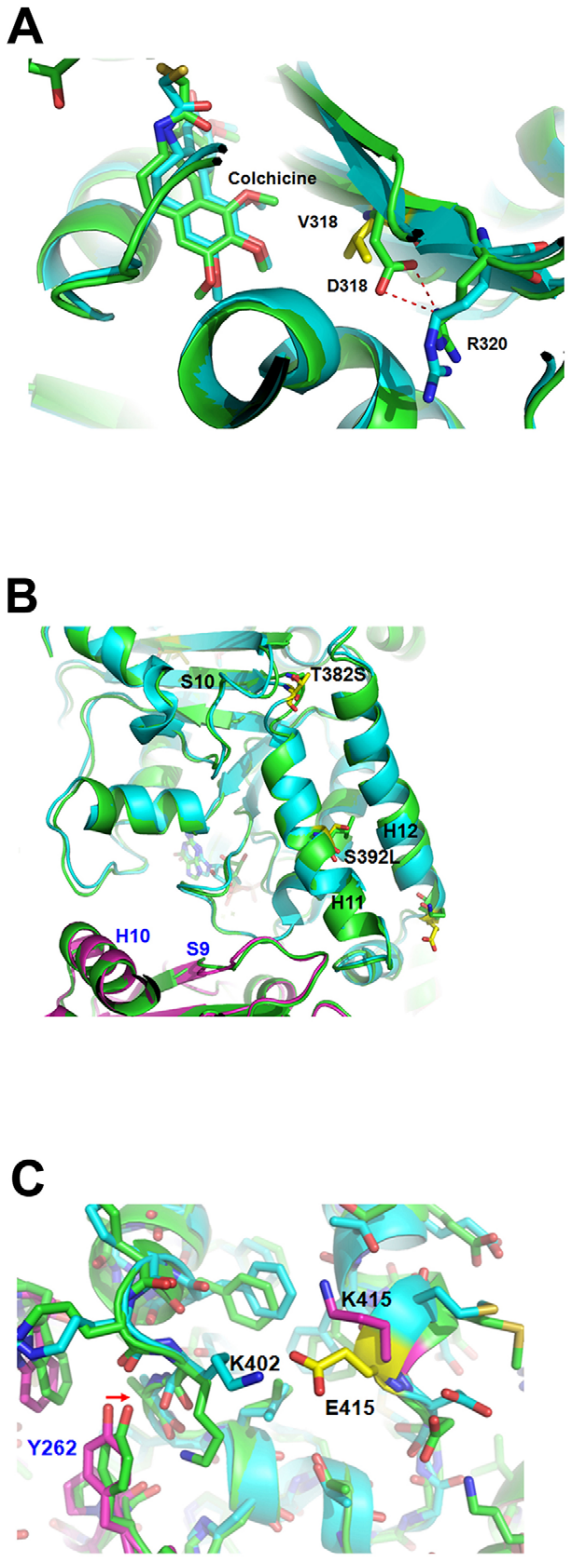
The computational modeling study indicates that tubulin mutations in KB-*L30* cells may increase microtubule assembly and stability (A, B and C).

Tubulin mutations can be associated with either altered drug-binding site or changes in microtubule stability. Here, computational modeling was employed to give structural insights. The three-dimensional tubulin structure (PDB code: 1SA0) was used [18] and the program Discovery Studio 2.1 (Accelrys, Inc) was applied to build model by mutating residues and performing energy minimization. In this model, the V318D residue (corresponding to V316D in KB-*L30*) is located in the colchicine-binding site on βI-tubulin (Figure 5A). Val318 (wild type) formed hydrophobic interactions with colchicine. However, the mutated residue (V mutated to D) shifts away from colchicine to form a hydrogen bond with residue R320, resulting in the loss of hydrophobic interaction with colchicine (Figure 5A) in the modeling study. This result indicates that V318D may weaken the binding affinity of colchicine to β-tubulin. On the other hand, mutations of residue 392 (located in Helix 11) and residue 382 (located in the loop near Helix 11) change the conformation of Helix 11, which might increase the longitudinal interactions and consequently alter the stability of microtubules (Figure 5B) [19]. E415K (corresponding to E405K in KB-*L30*) is located in the H11-H12 region near the dimer interface that is involved in longitudinal contacts. Residue 415, mutated from Glu to Lys, breaks the hydrogen-bond interactions with residue K402 and consequently leads to the movement of K402 closer to the α-tubulin. The shift of the K402 residue (Figure 5C) leads to close contacts with residues?V260, Y262 and W346 in the α-tubulin, increasing the interactions between β-tubulin and α-tubulin. Helix 11 is located in the interface of α-tubulin and β-tubulin dimer. In conclusion, the computational modeling study indicates that tubulin mutations in KB-*L30* cells may increase microtubule assembly and stability.

### KB-*L30* cells exhibit increased microtubule assembly and reduced microtubule dynamics

**Figure 6:**
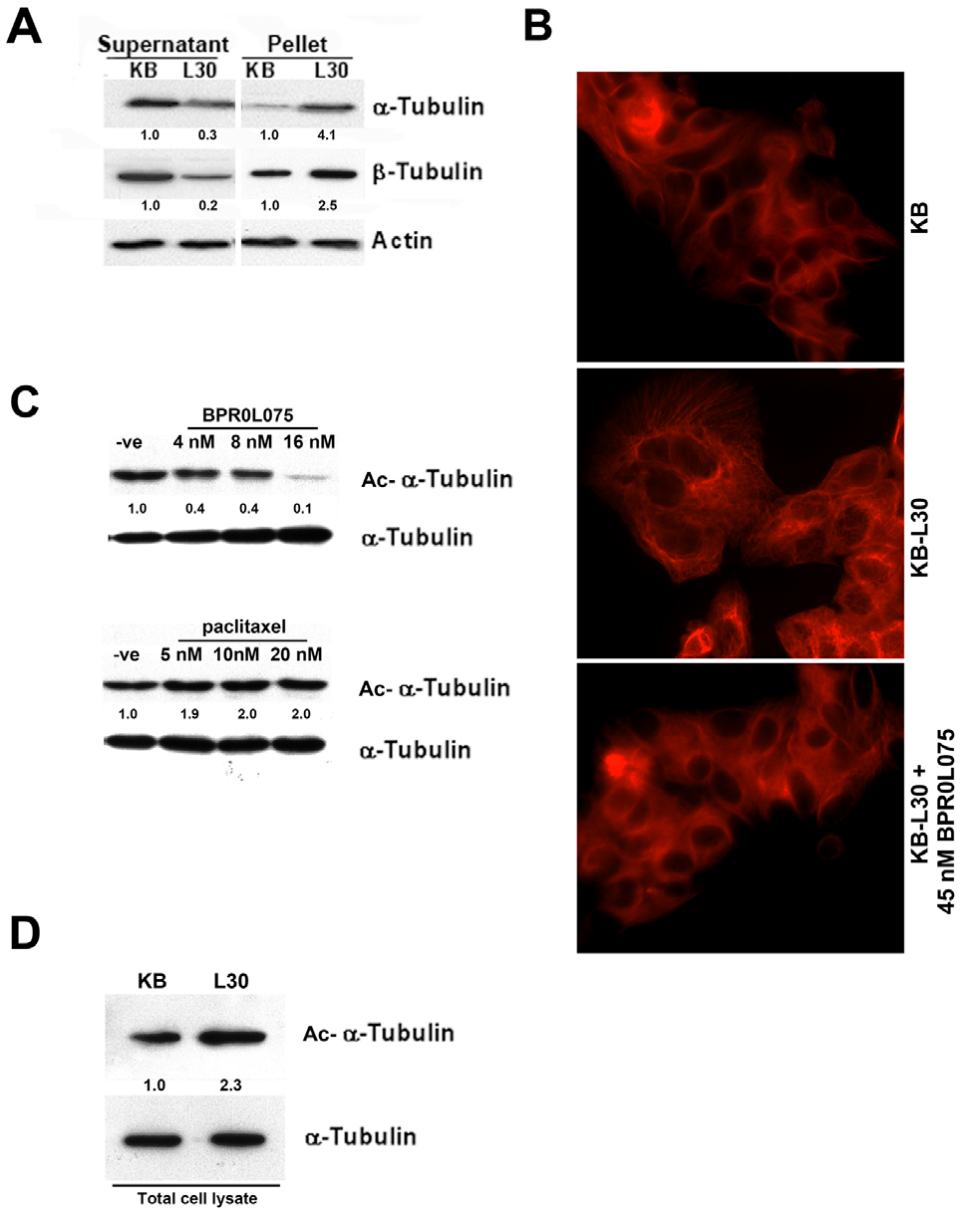
KB-*L30* cells exhibit increased microtubule assembly and reduced microtubule dynamics. The amount of soluble and insoluble α- and β-tubulin in KB and KB-*L30* cells was analyzed by Western blotting. Actin was used as a loading control (A). The integrity of microtubule networks in KB and KB-*L30* cells was showed by immuno-fluorescent microscopy. Cells were probed with anti-β-tubulin antibody (B). Microtubule dynamics in cells were analyzed by western blot analysis. Acetylated-α-tubulin was used as an indirect indicator of microtubule dynamics and the amount of acetylated-α-tubulin was analyzed in BPR0L075 and paciltaxel-treated KB cells by Western blotting. Alpha-tubulin (total form) was used as the loading control (C). The amount of the endogenously acetylated-α-tubulin in KB and KB-*L30* cells was analyzed by Western blotting. Alpha-tubulin (total form) was used as the loading control (D).

To determine whether the stability and dynamics of microtubule in KB-*L30* cells were affected by mutations in the βI-tubulin as predicated by computational analysis, Western blot analysis was performed. Here, Western blot analysis revealed that the amount both α- and β-tubulin pellets present in KB-*L30* cells were increased when compared to its parental KB cells (Figure 6A). In contrast, the soluble form of β-tubulin present in KB-*L30* cells was decreased compared to that of KB cells (Figure 6A). Immunofluorescence staining was also used to further support the above result. Microtubules were labeled with the rodamine-conjugated anti-β-tubulin antibody and cells were viewed under the microscope. KB-*L30* cells seem to have higher microtubule content as compared to KB cells (Figure 6B). In addition, the use of microtubule de-stabilizer, BPR0L075, was able to reduce the microtubule content in KB-*L30* cells (Figure 6B). Taken together, our results suggested that KB-*L30* cells exhibited increased microtubule assembly.

Microtubule dynamic instability is characterized by abrupt stochastic transitions between phases of extension and shortening. At the molecular level, increased α-tubulin acetylation is considered to be a marker of the reduced dynamic of microtubule. Here, Western blot analysis revealed that the amount of acetylated α-tubulin was decreased in BPR0L075-incubated KB cells in a concentration-dependent manner (Figure 6C). In contrast, the amount of acetylated α-tubulin was increased in paclitaxel-incubated cells (Figure 6C). Microtubule de-stabilizer was found functioning in inducing the microtubule dynamic instability and microtubule stabilizer was found functioning in reducing the microtubule dynamic instability [20], [21]. Thus, the amount of acetylated α-tubulin present in cells might be related to the sensitivity of cells to various anti-mitotic compounds. To determine whether KB-*L30* cells exhibited reduced microtubule dynamics, Western blot analysis was used to determine the level of α-tubulin acetylation present in the drug-resistant cells. Here, Western blot analysis revealed that the amount of acetylated α-tubulin was increased in KB-*L30* cells, as compared to KB cells (Figure 6D). Consistent with the prediction from the computational model, our results revealed that KB-*L30* cells exhibited both enhanced microtubule stabilization and reduced microtubule dynamics. Furthermore, our data indicate that the alteration of microtubule stability and dynamic in KB-*L30* cells may be caused by the presence of mutated βI-tubulin, resulting in the resistance to microtubule de-stabilizers and hyper-sensitivity to microtubule stabilizers.

## Discussion

Anti-mitotic compounds have been shown successful in treating various cancers. However, development of drug-resistance cells limits their efficacies in clinical situations. Therefore, it is important to determine possible drug resistance mechanism related to the application of anti-mitotic cancer therapy. Positive correlation between the expression of multi-drug resistance proteins MDR-1, MRP-1 and the induction of drug resistance to anti-mitotic compounds in cancers has been widely reported previously [4], [5]. However, drug resistance to anti-mitotic compounds in cancer cells is thought to be multi-factorial. Here, we demonstrated that the BPR0L075-resistant KB-*L30* cells do not express MDR-1 and MRP-1. Importantly, the current study indentifies novel sites of mutation in the exon 4 of βI-tubulin that are able to induce both drug resistance to microtubule de-stabilizers and hyper-sensitivity to microtubule-stabilizers through both alterations in the drug-binding site and microtubule stability.

The BPR0L075-resistant KB-*L30* cells used in this study seems to acquire its drug-resistant properties through MDR/MRP-independent mechanisms. Firstly, KB-*L30* cells did not express two of the most common drug efflux pumps, MDR-1 and MRP-1. In addition, unlike traditional microtubule inhibitors such as vincristine and paclitaxel, BPR0L075 is effective in suppressing cell growth of both MDR/MRP-positive and –negative tumor cell lines [14]. *In vivo*, BPR0L075 showed potent activity against the growth of xenograft tumors of the gastric carcinoma MKN-45, human cervical carcinoma KB, and KB-derived P-gp170/MDR -overexpressing KB-VIN10 cells in nude mice [14]. However, BPR0L075 was ineffective in inducing the death of KB-*L30* cells in this study. Therefore, our study indicated that MDR/MRP did not play an important role in the resistance to microtubule de-stabilizers such as BPR0L075, colchicine and vincristine in KB-*L30* cells.

Since MDR and MRP did not play an important role in the induction of drug resistance in KB-*L30* cells, other drug resistance mechanisms must be present in the cells. Tubulin-binding compounds such as vincristine, colchicine and paclitaxel, inhibit the progress of cell mitosis through the inhibition of the dynamic formation of mitotic spindle during G_2_/M phase. Therefore, mutations in the drug target, tubulin, may interfere with the effectiveness of anti-mitotic compounds. In fact, mutations in both α- and β-tubulin in Chinese hamster ovary cells have been shown to result in resistance to colchicine and vinblastine [8]. Furthermore, mutations in the β-tubulin in ovarian cancer cells have been shown to result in resistance to epothilone and taxanes [22]. In this study, six novel mutation sites were found present in the βI-tubulin gene of KB-*L30* cells. These mutations correspond to amino-acid mutations V316D, T372S, S382L, E405K, E432K and G437K. These mutation sites are presented in the exon four regions instead of exon one, two and three that have been previously identified [7]. This is particularly important as mutations in exon four in causing anti-mitotic drug resistance are less demonstrated when compared to exon 1, 2 and 3. In this study, over-expression of the mutant tubulins (contained multiple mutated-sites) in KB cells reduced the drug sensitivity to the microtubule de-stabilizing compound BPR0L075 as compared to cells transfected with empty vector or wildtype tubulin. The small differences between cells transfected with mutated-tubulin and wildtype tubulin were probably due to the large amount of the endogenously expressed wildtype tubulin present in the transfected KB cells. Transfection of constructs that contain β-tubulin with different single site mutation in KB cells was also performed to determine whether a specific single site mutation in the gene was indeed enough to cause drug resistance to BPR0L075 (data not shown). However, results of our study revealed no significant difference in drug sensitivity between cells transfected with the mutant (single site mutation) and wildtype βI-tubulin (data not shown). This result can possibly be explained by the followings: 1) the mutation sites were not directly located on the specific drug binding sites and 2) the three dimensional structure of βI-tubulin was not significantly altered by any single site mutation that was revealed in this study. Instead, all six mutations present on exon 4 might together contribute to the alteration of the βI-tubulin's three dimensional structures, resulting in the changes of drug sensitivity in KB-*L30* cells.

With the use of computational modeling, the mutant regions in the computational model which we predicted were analyzed to compare with the increase or decrease of the interactions before and after mutations. In our model, the locations of βI-tubulin mutations were identified and the resulting conformation changes in βI-tubulin protein were predicated with potential effects on microtubule assembly and stability. In agreement with the computational model, increased tubulin polymer levels were observed in the BPR0L075-resistant KB-*L30* cells as compared to its drug-sensitive parental cells (Figure 6A and 6B). In addition, the level of acetylated α-tubulin was also increased in KB-*L30* cells (Figure 6D), indicating that reduced microtubule dynamic instability was present in this specific cell line. A role for altered microtubule polymer levels in vincristine resistance of acute lymphoblastic leukemia has been demonstrated *in vivo* previously [23]. In addition, a vincristine-resistant xenograft with high levels of polymerized tubulin was shown relatively sensitive to the microtubule-polymerizing drug paclitaxel [23]. Furthermore, a study also reported that the dynamic instability of microtubules of paclitaxel-resistant A549-T12 cancer cells was significantly increased as compared to its parental cells [24]. Thus, enhanced microtubule polymerization and reduced dynamic instability caused by tubulin mutations may represent a survival mechanism against microtubule de-stabilizing compounds. On the other hand, the enhanced microtubule polymerization and reduced dynamic instability may improve the effectiveness of microtubule stabilizing compounds such as paclitaxel. In fact, our published study demonstrated that down-regulation of survivin (IAPs) induced microtubule de-polymerization, resulting in increased drug sensitivity of KB cells to colchicine and BPR0L075 [13]. In addition, the same treatment partially restored sensitivity of KB-*L30* cells to BPR0L075 through the process of microtubule de-polymerization [13]. However, down-regulation of survivin did not affect the activity of caspases [13]. Taken together, these results indicated that the balance between polymerized and non-polymerized tubulin may be an important determinant of response to anti-mitotic based chemotherapy.

It is also worth noting that the down-regulation of βII- and βIII-tubulin isotypes was observed in KB-*L30* cells. Literature revealed that the over-expression of βIII-tubulin induced paclitaxel- and docetaxel-resistance in cancer cells [7], [10], [16]. High level of βIII-tubulin expression was also shown to be associated with the resistance to paclitaxel and decreased survival in patients with carcinoma [25]. Interestingly, a recent study from Tseng *et al.* has provided a possible explanation for this phenomenon. Mathematical and computational models of their study indicate that microtubule de-stabilizing agents, colchincine and its derivatives, bind strongest to the βIII-tubulin as compare to other β-tubulin isotypes [26]. Thus, changes in the expression of βIII-tubulin can alter the sensitivity to colchicine derivatives that inhibit the polymerization of microtubules. Therefore, down-regulation of the βIII-tubulin may also contribute to the enhanced sensitivity to paclitaxel and the reduced sensitivity to colchicine/BPR0L075 in KB-*L30* cells.

In conclusion, we showed that novel mutations in exon 4 of the βI-tubulin induced resistance to microtubule de-stabilizers and hyper-sensitivity to microtubule stabilizer through alteration in the microtubule assembly and dynamics in cancer cells. This report further indicates that cancer cells may alter the stability and dynamics of microtubule networks through multiple mechanisms such as tubulin mutations and differential isotype expressions simultaneously, resulting in the resistant to anti-mitotic cancer therapy. The results of this study provide molecular information in drug selection for patients with specific tubulin mutations and also have important implications for the development of future anti-mitotic compounds that are able to target drug-resistant cancer cells. Nevertheless, further investigations are needed to determine whether these specific βI-tubulin mutations can be found in clinical samples (post-treatment) and its correlation to drug resistance.

## Materials and Methods

### Cell lines, antibodies and reagents

Human oral carcinoma cells (KB) were purchased from the American Type Culture Collection (ATCC, Manassas, VA). This specific cancer cell line was also described as a HeLa contaminant by ATCC. KB and KB-derived KB-*L30* cells were cultured in RPMI 1640 medium (Gibco, Grand Island, NY), supplemented with 5% fetal bovine serum, penicillin (100 U/mL), streptomycin (100 µg/mL) and L-glutamine (0.29 mg/mL), at 37°C. The antibodies used in this study included: a mouse anti-αtubulin antibody (Upstate Cell signaling, Lake Placid, NY), a mouse anti-βtubulin antibody (BD PharMingen, Franklin Lakes, NJ) and a mouse anti-actin antibody (Santa Cruz Biotechnology, Santa Cruz, CA).

### Establishment of the KB-derived BPR0L075-resistant cell line

Human oral carcinoma cells (KB) were cultured in RPMI 1640 medium as previously described. BPR0L075-resistant cells were established from KB cells by exposure to increasing concentrations of BPR0L075. Briefly, KB cells were initially incubated in completed medium containing 5 nM of BPR0L075 that yielded 40% cell survival for a period of 3∼4 weeks, and the cells that proliferated were repeatedly subcultured in completed medium containing increasing concentrations of the drug (at 20% increment each time). A mixed population of cells that grew exponentially in the present of 15 nM of BPR0L075 were obtained and further cultured in complete medium containing increasing concentrations of the drug to 20 nM of BPR0L075. Then, cells that grew exponentially in the present of 20 nM of BPR0L075 were obtained and subsequently cultured in complete medium containing increasing concentrations of the drug to 30 nM of BPR0L075. Cells that grew exponentially in the presence of 30 nM of BPR0L075 were obtained and subcloned by dilution plating in 48-well plates. Six individual clones were isolated. For maintenance, these subclones were cultured under conditions similar to those used for KB, except for the addition of BPR0L075 (30 nM). Since all of these subclones exhibited similar drug sensitivity to BPR0L075 and other microtubule de-stabilizers, only one subclone, KB-*L30*, was selected and further investigated in this study.

### RT-PCR

Total RNA was extracted with using TRIzol reagent (Invitrogen, Carlsbad, CA) and complementary DNA was synthesized from RNA with the SuperScriptTM First-Strand Synthesis System (Invitrogen, Carlsbad, CA). Expression levels of βI, βII, βIII, βIV_a_ and βIV_b_-tubulin transcript were determined by reverse transcriptase (RT)-polymerase chain reaction (PCR). Specific primers with following sequences were used: βI, βII, βIII, βIV_a_ and βIV_b_-tubulin common forward, 5′ CAACAGCACGGCCATCCAGG; βI-tubulin reverse, 5′ AAGGGGGCAGT TGAGTAAGACGG; βII-tubulin reverse, 5′ GTAGAAAGACCATGCTTGGG; βIII-tubulin reverse, 5′ CTTGGGGCC CTGGGCCTCCGA; βIV_a_-tubulin reverse, 5′ AAGTAGCCAGAGGTAAAGCGA and βIV_b_-tubulin reverse, 5′ CTTTCCCCAGTGACTGAAGG. To amplify different exons of βIII-tubulin, polymerase chain reaction was performed with target-specific primers that have been previously published [27] (Table 2) (Figure S1). PCR cycling conditions were as follows: an initial denaturing step at 95°C for 10 min, 40 cycles at 95°C for 30 sec, 50°C–58°C for 30 sec, 72°C for 30 sec, followed by a final period of extension at 72°C for 5 min. For amplification of the whole region of exon 4, the extension time was extended by 2 min.

**Table 2:** Different primers used in the PCR and SSCP analysis.

Primer		Sequence
**Exon 1**	Forward	*AACCTTCCAGCCTGCGAC*
	Reverse	*ACTTACCTGGATTTTTCCTTG*
**Exon 2**	Forward	*TAGTTGGGGACATAGTTGGC*
	Reverse	*TAAGGCGTGCCCAGAAATGG*
**Exon 3**	Forward	*AATGACAAGTCTCTGATCCC*
	Reverse	*TCCAATACAACAATCATCTCC*
**Exon 4**	Forward	*TGTATTGGAGTGCTAATACAG*
	Reverse	*CTCCCTTGAAGCTGAGATGG*
**Exon 4a**	Forward	*CATGTATCTTCCATACCCTG*
	Reverse	*CTGAAGGTATTCATGATGCG*
**Exon 4b**	Forward	*GAATGGGCACTCTCCTTATC*
	Reverse	*GGACCATGTTGACTGCCAAC*
**Exon 4c**	Forward	*ATGAGTGGTGTCACCACCTG*
	Reverse	*GACTGCCATCTTGAGGCCAC*
**Exon 4d**	Forward	*CCCAACAATGTCAAGACAGC*
	Reverse	*CAAGATAGAGGCAGCAAACAC*

All primer sequences are listed in the direction of 5′ to 3′.

### Single-stranded conformation polymorphism (SSCP) analysis

Genomic DNA was extracted from KB and KB-*L30* cells. SSCP analysis was performed using 5% nondenaturing polyacrylamide gels (acrylamide/N,N-bisacrylamide 99∶1) containing 10% glycerol. After electrophoresis for 4.5 h at 850 V, the gels were stained with SYBR Green II (TaKaRa, Otsu, Japan) and scanned with a fluorescence image analyzer (Alpha Innotech, San Leandro, CA).

### SDS-PAGE and Western Blot Analysis

Cells were lysed with ice-cold lysis buffer (10 mM Tris, 1 mM EDTA, 1 mM DTT, 60 mM KCl, 0.5% v/v NP-40 and protease inhibitors). Total cell lysates, fractions of supernatant or pellet were resolved on 10% and 12% polyacrylamide SDS gels under reducing conditions. The resolved proteins were electrophoretically transferred to PVDF membranes (Amersham Life Science, Amersham, U.K.) for Western blot analysis. The membranes were blocked with 5% non-fat milk powder at room temperature for two hours, washed twice with PBST (1% Tween) and then incubated with primary antibody for 90 minutes at room temperature. The membranes were washed twice with PBST then subsequently incubated with a horseradish peroxidase-conjugated secondary antibody (dilution at 1∶10000, Santa Cruz Biotechnology, Santa Cruz, CA). Immunoreactivity was detected by Enhanced Chemiluminescence (Amersham International, Buckingham, U.K.) and autoradiography.

### Computational modeling

We employed the program Discovery Studio 2.1 (Accelrys, Inc) to build the computational models of tubulin mutants. The three-dimensional structure of wild-type tubulin (PDB code: 1SA0) was used as a template to perform energy minimization. The force fields of conformations were further check with CHARMm, and the parameters used were set as default values.

## Supporting Information

Figure S1 Schematic diagram showing the process of PCR with different PCR primers.
(1.65 MB TIF)
