# Supplementary figures and images for: Cancer Cells Acquire Mitotic Drug Resistance Properties Through Beta I-Tubulin Mutations and Alterations in the Expression of Beta-Tubulin Isotypes
Source: PLoS One. 2010 Sep 3;5(9):e12564. doi: 10.1371/journal.pone.0012564 (PMC2933234; doi:10.1371/journal.pone.0012564)

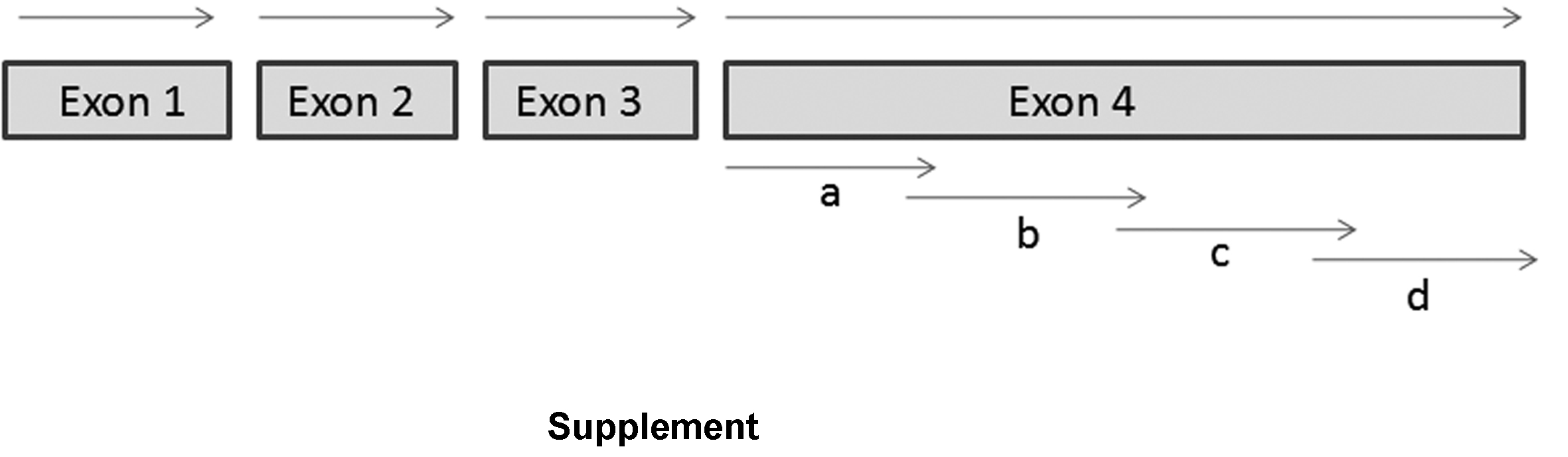

Supplement: Figure S1 — Schematic diagram showing the process of PCR with different PCR primers. (1.65 MB TIF) [file pone.0012564.s001.tif]
